# Chinese herbal injection for cardio-cerebrovascular disease: Overview and challenges

**DOI:** 10.3389/fphar.2023.1038906

**Published:** 2023-02-24

**Authors:** Jiang Huajuan, Huang Xulong, Xian Bin, Wang Yue, Zhou Yongfeng, Ren Chaoxiang, Pei Jin

**Affiliations:** ^1^ State Key Laboratory of Southwestern Chinese Medicine Resources, Chengdu, China; ^2^ Pharmacy College, Chengdu University of Traditional Chinese Medicine, Chengdu, China

**Keywords:** Chinese herbal injection, cardio-cerebrovascular diseases, clinical application, pharmacological effects, traditional Chinese medicine

## Abstract

Cardio-cerebrovascular diseases are the leading cause of death worldwide and there is currently no optimal treatment plan. Chinese herbal medicine injection (CHI) is obtained by combining traditional Chinese medicine (TCM) theory and modern production technology. It retains some characteristics of TCM while adding injection characteristics. CHI has played an important role in the treatment of critical diseases, especially cardio-cerebrovascular diseases, and has shown unique therapeutic advantages. TCMs that promote blood circulation and remove blood stasis, such as *Salvia miltiorrhiza*, *Carthami flos*, *Panax notoginseng*, and *Chuanxiong rhizoma*, account for a large proportion of CHIs of cardio-cerebrovascular disease. CHI is used to treat cardio-cerebrovascular diseases and has potential pharmacological activities such as anti-platelet aggregation, anti-inflammatory, anti-fibrosis, and anti-apoptosis. However, CHIs have changed the traditional method of administering TCMs, and the drugs directly enter the bloodstream, which may produce new pharmacological effects or adverse reactions. This article summarizes the clinical application, pharmacological effects, and mechanism of action of different varieties of CHIs commonly used in the treatment of cardio-cerebrovascular diseases, analyzes the causes of adverse reactions, and proposes suggestions for rational drug use and pharmaceutical care methods to provide a reference for the rational application of CHIs for cardio-cerebrovascular diseases.

## Introduction

Cardio-cerebrovascular disease is one of the most serious diseases that threaten human health today; its morbidity and mortality rates have surpassed those of tumor diseases and now rank first (Liberale et al., 2021; Xu et al., 2022). The heart and brain are the most closely related organs physiologically, and brain tissue relies on blood circulation driven by the heart to maintain normal physiological functions. At the same time, cardiovascular and cerebrovascular diseases are pathologically based on vascular occlusion caused by atherosclerotic rupture related to blood lipid levels. When abnormal blood rheology occurs, atherosclerosis may involve multiple organs, particularly the heart and brain (Novo et al., 2014; Novo et al., 2019; Keeter et al., 2022). Furthermore, cardio-cerebrovascular diseases have the same pathological basis. Research data show that about 10%–45% of patients with heart disease may have a stroke, about 78.1%–90.2% of patients with cerebrovascular disease have an abnormal electrocardiogram, and 12.7% can be complicated by cerebral infarction (Xu et al., 2021; Lu et al., 2022).

Although conventional Western medical treatments such as nitrates, statins, receptor blockers, clopidogrel, and aspirin have good effects on cardio-cerebrovascular diseases, there are still great risks, such as embolism and bleeding caused by excessive antithrombotics (Wang Y. et al., 2022; Sikora et al., 2022). Chinese herbal injections (CHIs) play an irreplaceable role in the treatment of cardio-cerebrovascular diseases and have economic and social benefits (Feng et al., 2021; Li et al., 2022b). An increasing number of doctors tend to use certain CHIs combined with conventional Western medicine to improve their therapeutic effect (Li et al., 2015a; Liu et al., 2016). As a new dosage form of traditional Chinese medicine (TCM) preparation, CHI not only has the characteristics of injection, but also retains the characteristics of TCM to a certain extent. Its active ingredients and modern pharmacological effects are clear, and it avoids degradation of the gastrointestinal tract and the first-pass effect of the liver following oral drug administration. The clinical application of CHI is more convenient, its effect is faster, and it plays an important role in the treatment of acute and severe diseases. The research and development of CHI varieties mainly focus on the treatment of cardio-cerebrovascular diseases, respiratory systems, and tumors; in particular, the therapeutic effect of cardio-cerebrovascular diseases has been recognized by doctors and patients. A total of 134 types of CHI have been listed, encompassing 158 types of raw materials for prescription decoction pieces, and the majority (56.7%) of medicinal materials are single-agent. The types and sales of CHI for the treatment of cardio-cerebrovascular diseases are the most numerous (Hao et al., 2020).

In terms of medicine composition, TCM to promote blood circulation and remove blood stasis accounts for a large proportion of CHI for cardio-cerebrovascular diseases, such as *Salvia miltiorrhiza*, *Carthami flos*, *Panax notoginseng*, and *Chuanxiong rhizoma* (Li et al., 2015a; Sun et al., 2022). Cardio-cerebrovascular diseases such as cerebral thrombosis and coronary heart disease are related to enhanced platelet function, blood thickening, and changes in hemodynamic characteristics (Gresele et al., 2021; Li et al., 2022c). In TCM, drugs that promote blood circulation and remove blood stasis dredge blood vessels and eliminate blood stasis, which can change the platelet function and hemodynamics of patients (Li D. et al., 2022). Patients with cardio-cerebrovascular diseases can use drugs to promote blood circulation and remove blood stasis to clear blocked blood vessels and improve blood supply. TCM for promoting blood circulation and removing blood stasis has unique advantages for the treatment of cardio-cerebrovascular diseases (Li et al., 2015a; Guo R. et al., 2020).

## CHI for cardio-cerebrovascular diseases

The CHI clinically used for the treatment of cardio-cerebrovascular diseases includes extracts obtained from TCM or the effective parts and single components obtained by further purification. TCM prescription injections are obtained by extracting and purifying TCM prescriptions based on the compatibility of TCM. Among these, *S. miltiorrhiza*, *C. flos*, *P. notoginseng*, and *C. rhizoma* are important drugs developed for the treatment of cardio-cerebrovascular diseases. The details of each CHI were obtained from the Chinese Medicine Information Query Platform (https://www.dayi.org.cn/), as shown in Table 1.

**TABLE 1:** CHIs for the treatment of cardio-cerebrovascular diseases.

Name	Composition of medicinal materials	Preparation process	Approved year	Main components	Clinical application	Pharmacological action	Dosage
Danhong injection (DHI)	*Salvia miltiorrhiza* Bunge [Lamiaceae; Salviae miltiorrhizae radix et rhizoma], *Carthamus tinctorius* L. [Compositae; Carthami Flos]	Soak Salviae miltiorrhizae radix et rhizoma twice in dilute ethanol and filter. Mix the dregs with Carthami Flos, add water, soak twice, combine the filtrate, add sodium chloride for injection to isotonicity, adjust pH to 6–7, filter, refrigerate for 24 h, add water for injection to the specified amount, filter, pot and sterilize	2002	Tanshinone IIA, danshensu, safflower yellow	Coronary heart disease, angina pectoris, myocardial infarction, blood stasis pulmonary heart disease, ischemic encephalopathy, cerebral thrombosis	Anti-inflammatory, antioxidant, anticoagulant, anti-apoptotic, protecting vascular endothelium, inhibiting platelet aggregation, reducing blood lipids	1. Intramuscular injection, 2–4 mL each dose, 1–2 times a day
							2. Intravenous injection, 4 mL each dose, 1–2 times a day
							3. Intravenous infusion, 20–40 mL each dose, 1–2 times a day
Honghua injection (HHI)	*Carthamus tinctorius* L. [Compositae; Carthami Flos]	Add water to Carthami Flos (500 g), and decoct three times. The concentrated solution is precipitated twice with ethanol, pH is adjusted with 50% sodium hydroxide solution. Then water is added for injection, after which it is filtered, potted, and sterilized	2012	Safflower yellow, kaempferol	Obliterative cerebrovascular disease, coronary heart disease, vasculitis	Anticoagulant, antiplatelet aggregation, coronary dilation	1. Treatment of occlusive cerebrovascular disease: intravenous drip, 15 mL each dose, once daily
							2. Treatment of coronary heart disease: intravenous drip. 5–20 mL each dose, once daily
							3. Treatment of vasculitis: intramuscular injection. 2.5–5 mL each dose, 1–2 times a day.
Safflower Yellow for Injection (SYI)	*Carthamus tinctorius* L. [Compositae; Carthami Flos]	Carthami Flos is extracted with water. The extract is concentrated and eluted by column chromatography, and the total safflower yellow is recovered as a solvent and concentrated and dried	2005	Hydroxysafflor yellow A; anhydrosafflor yellow B	Stable exertional angina	Inhibits arrhythmia, reduces infarct size, increases coronary blood flow, lowers blood pressure, slows heart rate, and reduces myocardial oxygen consumption	Intravenous infusion, 100 mg of safflower yellow for injection, added to 250 mL of 0.9% sodium chloride injection, intravenous infusion slowly, once daily; 14 days treatment course.
Danshen injection (DSI)	*Salvia miltiorrhiza* Bunge [Lamiaceae; Salviae miltiorrhizae radix et rhizoma]	Take 1,500 g of Salviae miltiorrhizae radix et rhizoma, add water and decoct three times, combine the decoction, concentrate, add ethanol to precipitate for two times, recover ethanol from the filtrate and concentrate to about 250 mL. Add water for injection to 400 mL and mix, adjust pH to 6.8 with 10% sodium hydroxide solution, boil for half an hour, filter, add water for injection to 1,000 mL and seal, sterilize.	2011	Tanshinone IIA and danshensu	Coronary heart disease, angina pectoris	The anticoagulant effect, promote fibrin degradation, improve myocardial ischemia, anti-atherosclerosis, anti-thrombotic	Intramuscular injection, 2–4 mL each dose, 1–2 times a day; intravenous injection, 4 mL each dose, 1–2 times a day; intravenous drip, 10–20 mL each dose, once daily
Xuesaitong injection (XSTI)	*Panax notoginseng* (Burkill) F.H.Chen [Araliaceae, Notoginseng radix et rhizoma]	Notoginseng radix et rhizoma is crushed into a coarse powder, extracted with 70% ethanol and filtered. The filtrate is concentrated under reduced pressure, filtered, passed through a column of styrene-type non-polar copolymer macroporous adsorbent resin, and washed with water. The aqueous eluate is discarded after which it is eluted with 80% ethanol. The eluate is concentrated under reduced pressure, decolorized, refined, concentrated under reduced pressure to infusion and dried	2001	Ginsenoside Rb1, ginsenoside Rg1, panax notoginsenosides	Atherothrombotic cerebral infarction, cerebral embolism, central retinal vein occlusion	Inhibit platelet aggregation and activation, antithrombotic, promote hematopoietic cell proliferation, lower blood lipids, and blood pressure, prevent atherosclerosis	1. Intramuscular injection: 100 mg once, 1–2 times daily
							2. Intravenous infusion: 200–400 mg each dose, once daily.
Shenmai Injection (SMI)	*Panax ginseng* C.A.Mey. [Araliaceae, Ginseng Radix et Rhizoma Rubra], *Ophiopogon japonicus* (L.f) Ker-Gawl. [Lillaceae; Ophiopogonis radix]	Red ginseng and Ophiopogonis radix are extracted twice with water, and the decoction is concentrated and added to a solution of 101 clarifiers at 7% of the volume of the solution, stirred well, and left for several hours to produce flocculation in the extract. Then an equal amount of suspension aid 5% suspension is added and stirred well, centrifuged, dispensed and sterilized	2010	Ginsenosides Rb1, Rg1, Re	Shock, coronary heart disease, viral myocarditis, chronic pulmonary heart disease, neutropenia	Inhibit cardiovascular oxidative stress, regulate calcium balance, improve mitochondrial function and inhibit apoptosis, inhibit neuronal apoptosis, and maintain blood-brain barrier integrity after cerebral ischemia	1. Intramuscular injection of 2–4 mL once daily
							2. Intravenous infusion, 20–100 mL each dose
Danshen Ligustrazine Injection (DLI)	*Salvia miltiorrhiza* Bunge [Lamiaceae; Salviae miltiorrhizae radix et rhizoma], *Ligusticum chuanxiong* Hort. [Apiaceae; Chuanxiong Rhizoma]	After taking Salviae miltiorrhizae radix et rhizoma by water extraction and treated using the stone sulfur method, ethanol is recovered by alcohol precipitation twice (the first time to make the alcohol content reach 60%, the second time to make the alcohol content reach 70%) to make a clear liquid containing 0.4 g of medicinal material per 1 mL. To adjust the pH value, as a backup solution, mix Chuanxiongzin hydrochloride, glycerol and the above solution evenly, add water for injection and adjust the pH value of the solution with a hydrochloric acid solution to make a total of 1,000 mL. The solution is filtered, sealed in 5 mL ampoule and sterilized (115°C, 30 min).	2002	Danshensu, ligustrazine hydrochloride	Obstructive cerebrovascular diseases, such as cerebral insufficiency, cerebral thrombosis, cerebral embolism, and other ischemic cardiovascular diseases, such as coronary heart disease, chest tightness, angina pectoris, myocardial infarction, ischemic stroke, thromboangiitis obliterans, etc.	Anti-platelet aggregation, dilate coronary arteries, reduce blood viscosity, accelerate the flow rate of red blood cells, improve microcirculation, and anti-myocardial ischemia and myocardial infarction effects	Intravenous infusion, diluted with 5%–10% glucose injection or normal saline 250–500 mL, 5–10 mL each dose
Puerarin Injection (PI)	*Pueraria* lobata (Willd.) Ohwi [Fabaceae; Radix Puerariae Lobatae]	Sterilized aqueous solution made of Puerarin with the appropriate amount of co-solvent.	2004	Puerarin	Coronary heart disease, angina pectoris, myocardial infarction, retinal artery, and vein occlusion, sudden deafness	Dilate coronary and cerebrovascular, reduce myocardial oxygen consumption, improve microcirculation and anti-platelet aggregation	Intravenous infusion, 200–400 mg each dose, add 250–500 mL of glucose injection for intravenous infusion, once daily, 10–20 days treatment course, can be used continuously for 2–3 courses of treatment
Huangqi Injection (HQI)	*Astragalus mongholicus* Bunge [Fabaceae; Astragali Radix]	Take 2000 g of Astragali Radix, add water and decoct three times, each time for 1.5 h, combine the decoction, filter, concentrate the filtrate, precipitate with ethanol twice, refrigerate each time, recover the ethanol and concentrate, dilute with water for injection, refrigerate for 12 h, filter, concentrate the filtrate, let it cool, adjust the pH to 7.5 with 20% sodium hydroxide solution, boil, add 0.125% activated carbon, boil for 5 min, filter while hot, add water for injection to make 1,000 mL, filter, adjust the pH to 7.5 with 20% sodium hydroxide solution, filter, pot, sterilize. Add 0.125% activated carbon, boil for 5 min, filter out while hot, add water for injection to make 1,000 mL, filter out, then adjust pH to 7.5 with 20% sodium hydroxide solution, filter out, pot and sterilize	2010	Astragaloside IV	Viral myocarditis due to heart qi deficiency and blood stasis, heart insufficiency and hepatitis due to spleen deficiency and dampness	It has a positive inotropic effect on the heart, enhances myocardial contractility, increases coronary blood flow, protects myocardial cells, and improves cardiovascular function	Intramuscular injection, 2–4 mL each dose, 1–2 times daily. Intravenous infusion, 10–20 mL each dose, once daily.
Mailuoning Injection (MLNI)	*Achyranthes bidentata* Blume [Amaranthaceae; Achyranthis Bidentatae Radix]	The decoction is concentrated into semi-solid form and precipitated with ethanol, the alcoholic solution is recovered and extracted with ethyl acetate, and the extract is dissolved in distilled water, the aqueous solution is filtered with an appropriate amount of Tween 80% and 20% NaOH solution to adjust its pH value to 8.5–9.0, filtered, potted, and sterilized	2011	Artesinolide	Thromboangiitis obliterans, arteriosclerotic obliterans, cerebral thrombosis and sequelae, venous thrombosis	It can dilate small blood vessels, coronary arteries, and veins, increase vascular perfusion, enhance myocardial contractility, improve blood circulation, increase fibrinolytic activity, and improve blood rheology	Intravenous infusion, 10–20 mL each dose, once daily, 10–14 days treatment course, severe patients can use 2–3 courses of treatment continuously
	*Scrophularia ningpoensis* Hemsl. [Scrophulariaceae; Scrophulariae Radix], *Dendrobium nobile* Lindl. [Orchidaceae; Dendrobii Caulis]						
	*Lonicera japonica* Thunb. [Caprifoliaceae; Lonicerae japonicae flos]						
Xingnaojing injection (XNJI)	Artificial Moschus, *Gardenia jasminoides* Ellis [Rubiaceae; Gardeniae fructus], Borneolum syntheticum	Add water of about 1,500 mL to Curcumae radix and Gardeniae fructus for distillation, collect distillate (1,000 mL), add musk into the above distillate, distill, collect distillate (1,000 mL). Take Borneolum syntheticum and add 8 g of polysorbate 80, mix well, add into distillate, mix well, add 8 g of sodium chloride, stir to dissolve, mix well, filter, pot, and sterilize	2003	Muskone	Cerebral embolism, acute cerebral hemorrhage, craniocerebral trauma, acute alcoholism with the above symptoms	Improve the permeability of the blood-brain barrier, protect the structure of the blood-brain barrier, inhibit inflammation, reduce cerebral ischemia-reperfusion injury, and improve neurological function	1. Intramuscular injection, 2–4 mL each dose, 1–2 times a day
							2. Intravenous infusion, 10–20 mL each dose
Xinmailong injection (XMLI)	*Periplaneta americana* (L.)	Dried *Periplaneta americana* powder is refluxed with 95% ethanol at 80°C for 1 h and filtered. The filtrate is concentrated to dryness under reduced pressure and the resulting extract is warmed and dissolved in 50 mL of distilled water and filtered. 5 mL of the filtrate is extracted and treated with strong alkaline anion exchange resin 717	2006	L-tryptophan, L-tyrosine, N-acetyldopamine	Adjuvant medication for chronic congestive heart failure caused by chronic pulmonary heart disease	Strengthen the heart, improve myocardial cell energy supply, expand coronary artery, increase coronary blood flow, inhibit oxygen free radicals mediated muscle damage, anti-arrhythmia	Each dose 5 mg/kg body weight, intravenous infusion, twice daily, with an interval of more than 6 h between the two doses. 5 days treatment course
Dazhu Hongjingtian injection (HJTI)	*Rhodiola wallichiana* (Hook.) S. H. Fu var. cholaensis (Praeg.) S.H.Fu [Crassulaceae; Rhodiola wallichiana var. cholaensis]	Take 1,670 g of Rhodiola wallichiana var. cholaensis, add water, and decoct three times, combine the decoction, and filter through. The filtrate is concentrated, ethanol is added to make the alcohol content reach 70%, it is stirred well, refrigerated for 24 h, filtered, the filtrate is combined, ethanol is recovered and concentrated, ethanol is added to make the content reach 85%, filtered, the filtrate is adjusted to pH 7.0, activated carbon is added and boiled for 30 min, refrigerated for 72 h, the filtrate is filtered by removing the carbon, the filtrate is filtered by ultra-column with a cut-off molecular weight of 10,000 g/mol. The filtrate is ultrafiltered by ultra-column with a cut-off molecular weight of 10,000, freeze-dried, and the lyophilized material is prepared by adding water for injection to 1,000 mL, filtered by microporous membrane, potted and sterilized	2006	Gallic acid, syringic acid, salidroside	Coronary heart disease stable angina pectoris	Reduced peripheral vascular resistance and coronary resistance, increased coronary blood flow, decreased myocardial oxygen consumption; decreased platelet aggregation rate; decreased whole blood viscosity and plasma viscosity	Intravenous infusion, 10 mL each dose, once daily. 10 days treatment course
Ginkgo Damo injection (GDI)	*Ginkgo biloba* L. [Ginkgoaceae; Ginkgo folium]	Take dipyridamole, dissolve in deionized water, dissolve evenly, add hydrochloric acid dropwise to adjust pH = 3–6, and centrifuge. Dissolve in 20%–90% ethanol, add activated carbon, raise the temperature to 50°C–80°C, stir for 0.5–3 h, filter and decarbonize, then add hydrochloric acid to adjust pH = 3–6.	2002	Ginkgo total flavonoids, dipyridamole	Coronary heart disease, thromboembolic disease	Dilate coronary blood vessels and cerebral blood vessels, improve symptoms and memory function of cerebral ischemia; inhibit platelet aggregation and platelet release	Intravenous infusion, adults take 10–25 mL each dose, twice daily
		Mix dipyridamole and Ginkgo biloba extract and sterilize					
Shuxuening injection (SXNI)	*Ginkgo biloba* L. [Ginkgoaceae; Ginkgo folium]	Add anhydrous ethanol to the ginkgo extract, then dilute with 2000–3,000 mL of water for injection. Add antioxidant and pH adjuster, add activated carbon for needles, keep warm and stir for 10 min, decarbonize and filter, filter through a microporous membrane, add water for injection to 3,000–6,000 mL in filtrate, and sterilize	2004	Ginkgolide A, quercetin, isorhamnetin	Ischemic cardiovascular and cerebrovascular diseases, coronary heart disease, angina pectoris, cerebral embolism, cerebral vasospasm	Dilate blood vessels and improve microcirculation	Intramuscular injection, 10 mL at a time, 1–2 times daily. Intravenous infusion, 20 mL per day
Salvia miltiorrhiza polyphenolate for injection (SLI)	*Salvia miltiorrhiza* Bunge [Lamiaceae; Salviae miltiorrhizae radix et rhizoma]	After the crushing of Salviae miltiorrhizae radix et rhizoma, it is extracted using hot water, filtered, and concentrated. The filtrate is adsorbed using macroporous resin and washed with water to remove impurities, the polyphenolic acid salt adsorbed on the macroporous resin is eluted with aqueous low-grade alcohol, the eluate is concentrated to a certain volume under reduced pressure and added to anhydrous ethanol alcohol precipitation, the supernatant is poured out and the precipitate is discarded, dried and crushed to obtain Salvia miltiorrhiza polyphenolate	2005	Salvia Polyphenolate	Coronary heart disease stable angina pectoris	Inhibit platelet aggregation, inhibit thrombosis	Intravenous infusion, 200 mg each dose, once daily, 2 weeks treatment course
Shenxiong Glucose Injection (SXI)	*Salvia miltiorrhiza* Bunge [Lamiaceae; Salviae miltiorrhizae radix et rhizoma], *Ligusticum chuanxiong* Hort. [Apiaceae; Chuanxiong Rhizoma]	Salviae miltiorrhizae radix et rhizoma is extracted using water and treated using the rock-sulfur method, then alcohol is used twice to recover ethanol and adjust the pH as a backup solution. The filtrate is mixed with ligustrazine and added to the water for injection, and the pH value of the solution is adjusted with hydrochloric acid	2002	Danshensu, Ligustrazine Hydrochloride	Occlusive cerebrovascular disease and other ischemic vascular diseases	Anti-platelet aggregation, dilate coronary arteries, reduce blood viscosity, accelerate the flow rate of red blood cells, improve microcirculation, and anti-myocardial ischemia and myocardial infarction	Intravenous infusion, once daily, 100–200 mL each dose
Dengzhan Xixin injection (DZXXI)	*Erigeron breviscapus* (Vant.) Hand.-Mazz. [Compositae; Erigerontis Herba]	Boil a decoction of Erigerontis Herba twice with water, combine decoction, filter, concentrate under reduced pressure, add ethanol until the alcohol content reaches 80%, filter, the filtrate is recovered ethanol under reduced pressure and concentrated, extracted with ethyl acetate shaking, the extract is concentrated under reduced pressure, the determination of the total flavonoid content. Take the appropriate amount of extract (containing 4.5 g of total flavonoids), add water for injection to dissolve, adjust pH to 8–8.5 with 5 mol/L sodium hydroxide solution, add water for injection and 0.1% activated carbon for injection, heat and boil for 30 min, filter, add 8 g of sodium chloride for injection and dissolve, add water for injection to 1,000 mL, filter, pot and sterilize	2015	Astragaloside, total caffeate	Chest pain, ischemic stroke, coronary heart disease angina pectoris	Inhibits oxygen free radicals in the body, increases the content of reducing substances and exerts an antioxidant effect; it can reduce blood viscosity and protect cell membranes, thereby reducing blood LPO and increasing SOD content	1. Intravenous injection, 20–40 mL each dose, 1–2 times a day
							2. Intramuscular injection, 4 mL each dose, 2–3 times a day
Shengmai injection (SGMI)	*Panax ginseng* C.A.Mey. [Araliaceae; Ginseng Radix et Rhizoma Rubra], *Ophiopogon japonicus* (L.f) Ker-Gawl. [Lillaceae	Crush Ginseng Radix et Rhizoma Rubra into fine grains, extract with ethanol reflux 4–5 times, control the end point of extraction by thin layer method, combine the extracts, concentrate to a thick paste, add a sufficient quantity of water to 400 mL, stir well, refrigerate, filter through, filtrate for liquid preparation; collect 150 mL of distillate of Schisandrae Chinensis Fructus by water distillation, refrigerate, for liquid preparation, decoct the dregs with water three times, combine the decoctions and concentrate to a thick paste, add ethanol and concentrate to a thick paste, add water for injection to 200 mL, boil the filtrate with an appropriate amount of activated carbon for 30 min, cool slightly, filter to clarify for liquid preparation; make about 200 mL of a clear aqueous solution of maidenhair according to the preparation method of an aqueous solution of schisandra for liquid preparation. The above solution is mixed well, filtered, and the filtrate is added with water for injection to 1,000 mL, and the pH of the solution is adjusted to 7.5, filtered, potted, and sterilized.	2011	Ginsenosides Rb1, Rg1, Re, schisandrin A	Myocardial infarction, cardiogenic shock; septic shock	Improve microcirculation and anti-shock, reduce blood viscosity, anti-platelet aggregation	1. Intramuscular injection: 2–4 mL each dose, 1–2 times a day
	Ophiopogonis radix], *Schisandra chinensis* (Turcz.) Baill. [Magnoliaceae; Schisandrae Chinensis Fructus]						2. Intravenous infusion; 20–60 mL each dose

## Single TCM extract injection

CHI is prepared from a single component extracted and purified from Chinese herbal medicines such as PI. Puerarin is an isoflavone derivative with a crown expanding effect isolated from *Pueraria lobata radix*. Puerarin can be used to treat coronary heart disease, angina pectoris, and hypertension (Ma et al., 2022; Shao et al., 2022). Puerarin treatment causes an expansion of coronary blood vessels and cerebral blood vessels, improves local blood flow, improves microcirculation, inhibits platelet aggregation, reduces muscle oxygen consumption, and increases oxygen supply (Zeng et al., 2021; Chen D. et al., 2022; Lv et al., 2022).

**FIGURE 1:**
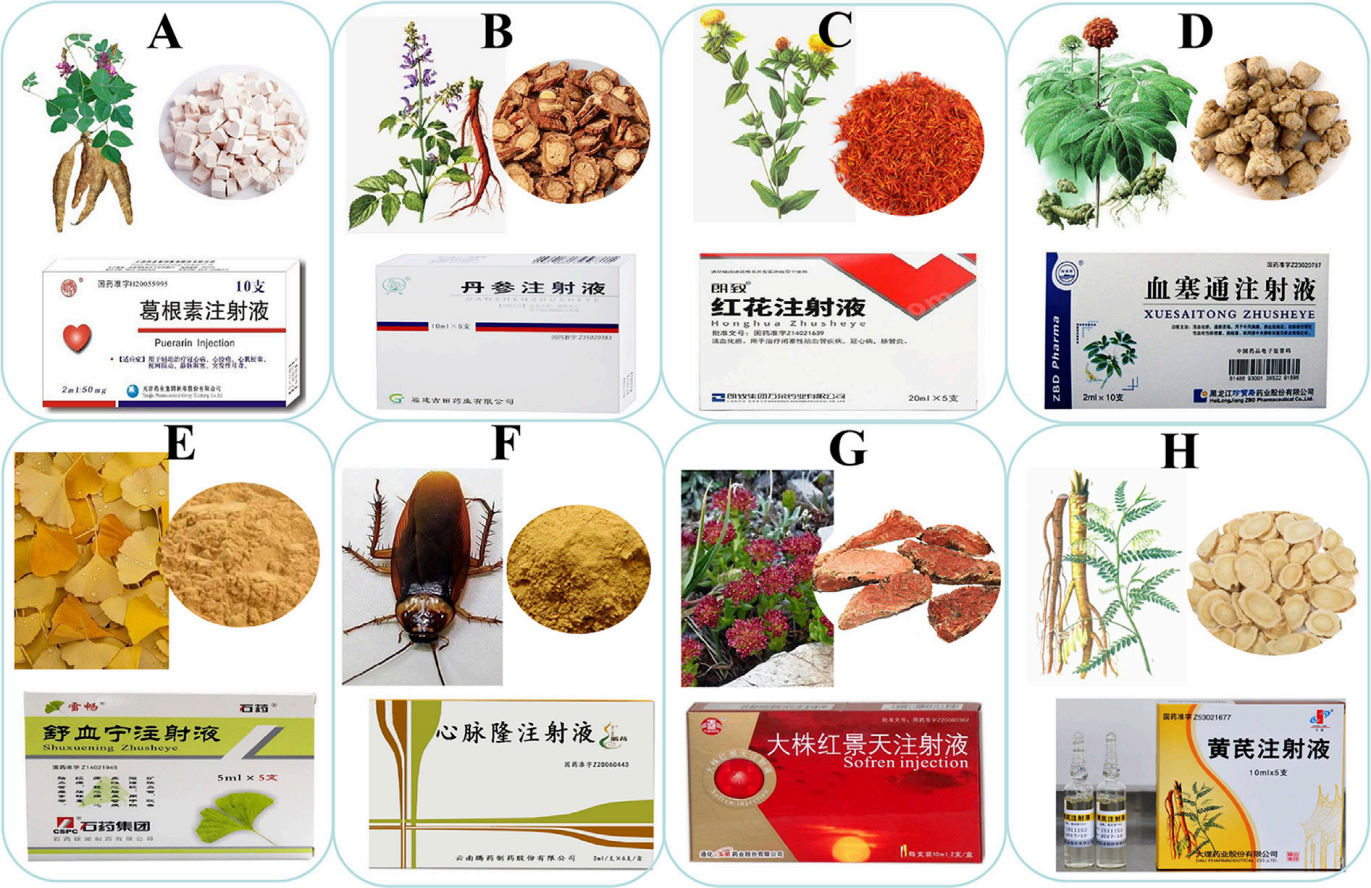
Single TCM extract injection; **(A)** Puerarin Injection; **(B)** Danshen injection; **(C)** Honghua injection; **(D)** Xuesaitong injection; **(E)** Shuxuening injection; **(F)** Xinmailong injection; **(G)** Dazhu Hongjingtian injection; **(H)** Huangqi injection.

CHI can be prepared from a single TCM extraction mixture, such as DSI, HHI, HQI, or DZXXI, as shown in Figure 1. DSI and SLI were prepared by extraction and purification of *S. miltiorrhiza*. *Salvia miltiorrhiza* promotes blood circulation, regulates menstruation, removes blood stasis, relieves pain, cools the blood, eliminates carbuncle, eliminates trouble, and soothes the nerves (Li H. et al., 2021; Wang SM. et al., 2022). Its main chemical components are danshensu, salvianolic acid, and tanshinone IIA, which can inhibit the activity of various coagulation factors and stimulate the plasmin system, thereby reducing thrombus formation and improving microcirculation. At the same time, *S. miltiorrhiza* has a good preventive effect on blood lipid metabolism by reducing the content of denatured lipoprotein, thereby preventing the development of atherosclerosis and reducing the incidence of cerebrovascular disease (Wang ZY. et al., 2022). *Salvia miltiorrhiza* can also reduce the content of oxygen free radicals produced by cardiovascular and cerebrovascular diseases, thereby protecting myocardial cells and brain tissue. In particular, Tanshinone IIA can increase the residence time of drugs in brain tissue (Zhong et al., 2021).

HHI and SYI were extracted and purified from the safflower. Safflower promotes blood circulation, removes stasis, relieves pain, and detoxifies blood. *Salvia miltiorrhiza* and safflower are essential medicines for promoting blood circulation and removing blood stasis (Orgah et al., 2020; Zhao et al., 2022). The main components of safflower are flavonoids, which are classified as quinoid chalcones, represented by hydroxysafflor yellow A, and common flavonoids, represented by kaempferol. The SYI was prepared by extracting the effective parts of the safflower. Safflower yellow can block platelet-activating factors, inhibit the release of serotonin, reduce peripheral resistance of blood vessels, expand cardiovascular and cerebrovascular vessels, inhibit platelet aggregation, significantly reduce blood viscosity and plasma viscosity, and improve the erythrocyte aggregation index; thus, blood platelet aggregation in the stasis model was reduced, and microcirculation disturbance was significantly improved. Prothrombin time following treatment with safflower yellow was delayed (Wang L. et al., 2021).

XSTI were prepared from the total saponins of *Panax notoginseng*. *Panax notoginseng* promotes blood circulation, removes blood stasis, reduces swelling and calms pain, promotes hemostasis, and is nourishing. It is the main drug used to treat traumatic injuries (Wang et al., 2016; Yang et al., 2018). *Panax notoginseng* saponins have a wide range of effects, such as dilation of the coronary arteries, improvement of left ventricular diastolic function, and reduction the concentration of Ca^2+^ in cardiomyocytes. It can also inhibit platelet aggregation and promote fibrinolysis (Duan et al., 2018; Xu CC. et al., 2019).

SXNI was prepared from Ginkgo leaf extract and GDI was prepared by mixing *Ginkgo biloba* L. leaf extract and dipyridamole. *Ginkgo biloba* L. leaves contain active ingredients such as flavonoid glycosides and terpenoid lactones, including quercetin, kaempferol, bilobalide, and ginkgolide, which have functions such as promoting blood circulation and remove blood stasis, dredge collaterals, relieve pain, astringe the lungs, relieve asthma, reduce turbidity, and lower lipids (Tian et al., 2017; Liu et al., 2021). *Ginkgo biloba* L*.* leaves can be used for blood stasis blocking collaterals, chest pain and heart pain, stroke hemiplegia, lung deficiency, cough and asthma, and hyperlipidemia (Wang et al., 2021a; Li et al., 2021d; Liang et al., 2022). *Ginkgo biloba* L. leaves can effectively inhibit platelet-activating factors, abnormal platelet aggregation, and thrombosis and reduce blood lipids and viscosity (Sarkar et al., 2020; Li R. et al., 2021).

XMLI was prepared from the extract of the animal TCM *Periplaneta americana*. *Periplaneta americana* is a natural animal medicine that can eliminate inflammation and edema, promote wound healing, and improve immune function (Lin et al., 2019; Zeng et al., 2019; Liao et al., 2022; Pang et al., 2022). HJTI is prepared from an extract of the traditional Chinese medicine *Rhodiolae Crenulatae Radix et Rhizoma*. *Dazhu Rhodiolae* has pharmacological effects including protection of the heart and nerves, anti-fatigue, anti-aging, anti-radiation, and immune regulation (Fan et al., 2020; Li et al., 2021c; Chen Y. et al., 2022).

## TCM prescription injection

**FIGURE 2:**
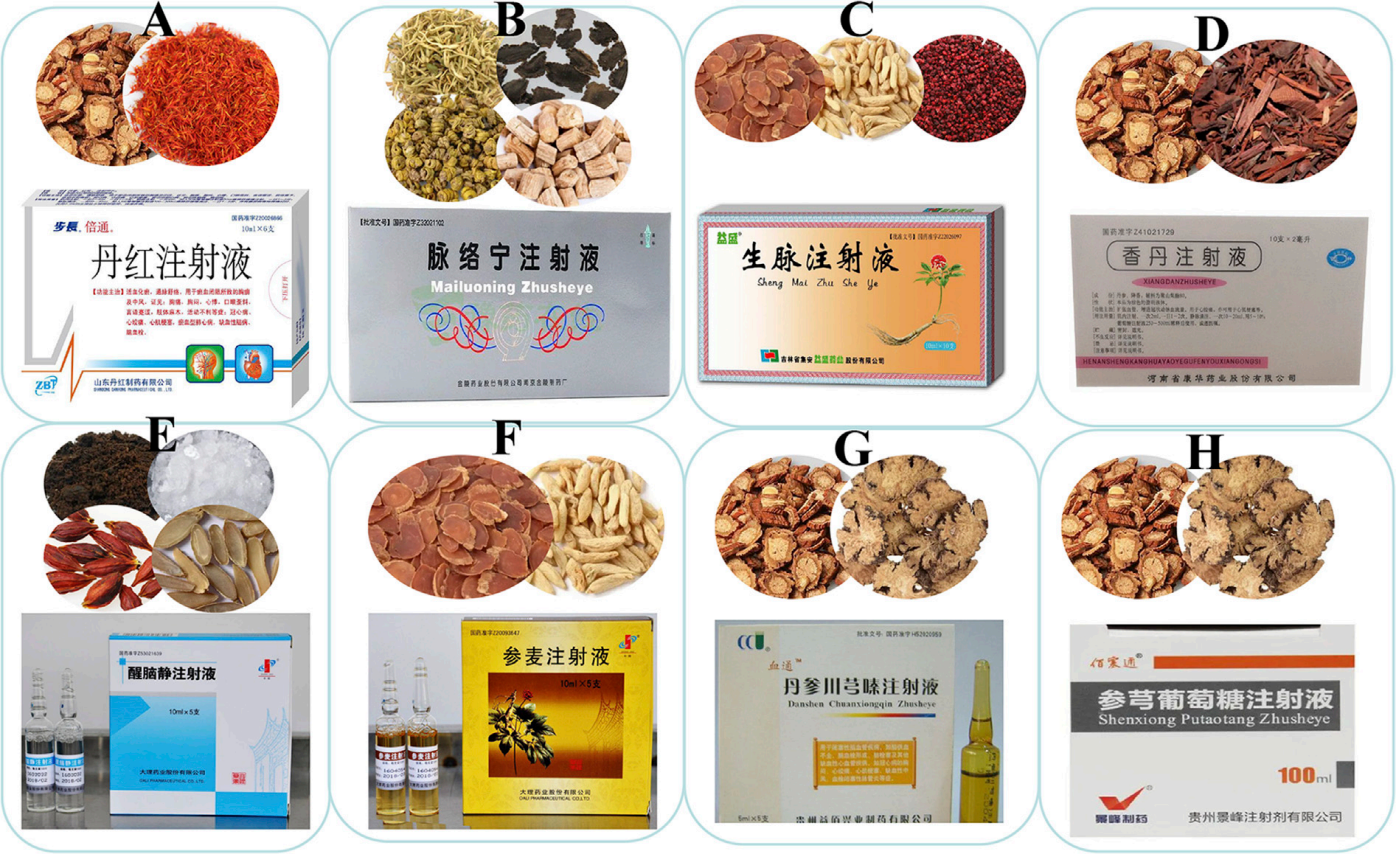
TCM Prescription Injection; **(A)** Danhong injection; **(B)** Mailuoning Injection; **(C)** Shengmai Injection; **(D)** Xiangdan injection; **(E)** Xingnaojing injection; **(F)** Shenmai injection; **(G)** Danshen Ligustrazine Injection; **(H)** Shenxiong Glucose Injection.

The compatibility of TCM prescriptions to reduce toxicity and increase efficacy is a characteristic of the clinical application of TCM. Based on the compatibility and combination characteristics of TCM prescriptions, a batch of TCM prescription injections was innovatively developed, as shown in Figure 2. Danhong injection (DHI) is obtained by water extraction and alcohol precipitation of two medicinal materials, *S. miltiorrhiza* and safflower. *Salvia miltiorrhiza* and safflower are currently widely used as clinical medicines to promote blood circulation and remove blood stasis (Li M. et al., 2018). SMI was derived from Shendongyin in “Zhengyin Maizhi,” written by Qin Jingming during the Ming Dynasty. It is a TCM preparation composed of *red ginseng* and *Ophiopogonis radix*. It nourishes qi, removes qi, nourishes yin, promotes body fluids, and nourishes blood vessels (Yu JH. et al., 2019; Xu HM. et al., 2019). MLNI is a TCM prescription developed based on the classic medical prescription “Si Miao Yong An San” in “Yanfang Xinbian,” guided by integrated traditional Chinese and Western medicine. It comprises *Lonicerae japonicae flos*, *Scrophulariae radix*, *Dendrobii caulis*, and *Achyranthis bidentatae radix*. Its functions include clearing heat, nourishing yin, promoting blood circulation, and removing blood stasis (Wang and Tian, 2014; Yang et al., 2015). XNJI was extracted from the Angong Niuhuang Pill, a classic first-aid prescription for stroke. It mainly consists of several TCM such as *Moschus*, *Borneolum syntheticum*, and *Gardeniae Fructus*. It is the only CHI approved in China for the treatment of acute cerebral hemorrhage and acute ischemic stroke in ambulances (Wang L. et al., 2022; Liu et al., 2022).

The DLI is one of the most commonly used injections in clinical practice. Unlike the traditional compatibility method, this injection combines the extract of *S. miltiorrhiza* with the active ingredients of *Ligusticum chuanxiong* (Xie et al., 2021; Ye et al., 2021). CHI is composed of the main active ingredients of these two medicinal materials. For example, SXI is composed of ligustrazine hydrochloride, the active ingredient of Chuanxiong, and Danshensu, the active ingredient of *S. miltiorrhiza* (Lu et al., 2020; Lu et al., 2021). Based on the classic drug pair theory of TCM, CHIs with effective active ingredients have been gradually developed. The injection obtained by combining the active ingredients has the advantages of a defined chemical composition, an evident pharmacological mechanism, and clinical indications.

## Pharmacological effects of Chinese herbal injection in the cardio-cerebrovascular diseases

**FIGURE 3:**
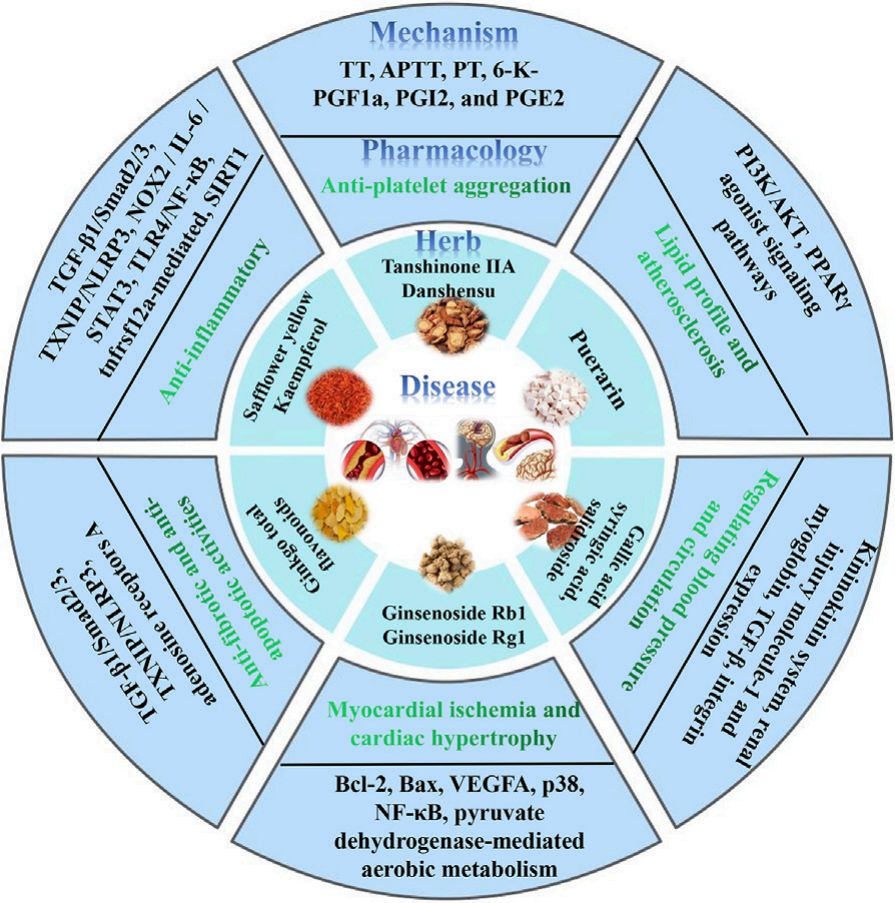
Data visualization of some Chinese herbal medicines, compounds, pharmacological effects, and mechanisms for the treatment of cardio-cerebrovascular diseases.

In recent years, based on the clinical application of CHIs, researchers have revealed their efficacy and mechanism of action through *in vitro* and *in vivo* pharmacological studies. Numerous studies have shown that CHIs for the treatment of cardio-cerebrovascular diseases have potential pharmacological activities, such as anti-platelet aggregation, anti-inflammatory, anti-fibrosis, and anti-apoptosis activities (Table 2; Figure 3).

**TABLE 2:** Pharmacological effects of CHIs in the cardio-cerebrovascular diseases.

Diseases	Type of study	Drug	Experimental model	Dose range tested	Duration	Results	References
Ischemic stroke	*In vivo*	GHI	I/R injury	2.5, 5, 10 mL/kg	0, 6, 23 h	(−) NO; iNOS; MPO; IL-1β; TNF-α; CRP; ICAM-1; NF-κB p65	Ai et al. (2017)
Ischemic heart disease	*In vitro*	SMI + DSI	Hypoxia/reoxygenation and H_2_O_2_-induced cardiomyocyte injury	2.5, 5 and 10 μL/mL	10 h	(+) cell viability; ΔΨm; PI3K/Akt; Erk1/2	Li et al. (2019b)
						(−) CK; LDH; ROS; Ca^2+^; cardiomyocyte injury	
Ischemic heart disease	*In vitro*	SMI	H9c2 cardiomyocytes were subjected to 12 h of hypoxia	1, 2.5, 5 μL/mL	24 h	(+) cell survival; ΔΨm; LC3, beclin 1, Parkin; Pink	Yu et al. (2019a)
						(−) mitochondrial mass and cytosolic Ca^2+^; mPTP opening; impaired mitochondrial respiration	
Ischemic heart disease	*In vitro*	SMI	Myocardial cells following I/R	5 mL/L	24 h	(+) Ca^2+^	Ye et al. (2015)
						(−) phosphorylated PLB; SERCA; aberrant apoptosis	
Cardiac toxicity induced by doxorubicin	*in vivo*	SMI	DOX-induced myocardial injury in C57BL/6 mice	2.5 mL/kg	6 days	(+) PI3K; p-Akt; p-GSK-3b; the ratio of L-OPA1 to S-OPA1; AMPK phosphorylation; DRP1	Li et al. (2020a)
						phosphorylation (−) mortality rate; levels of creatine kinase; creatine kinase-MB; Bax/Bcl-2; cleaved-Caspase3	
Stroke	*in vivo*	SMI	Middle cerebral artery occlusion (MCAO) rats	5 mL/kg	60 min after MCAO	(−) extravasation of FITC-albumin	Xu et al. (2019b)
						(+) flotillin-1; the translocation of occludin	
Myocardial infarction	*In vitro*	DLI	I/R and H/R	6.8, 20.4, 61.2 mg/kg	3 days	(+) cardiac function; Bcl-2/Bax ratio; Akt-eNOS	Huang et al. (2016)
						(−) myocardial infarct size; creatine kinase; lactate dehydrogenase; malondialdehyde levels; activation of caspase-3	
Stroke	*in vivo*	SLI	T1DM + MCAO rats	10.5, 21, 42 mg/kg	3 days	(+) brain microvasculature in ipsilateral; glucose uptake in the cortex; hippocampus; penumbra; HQ-1; HQO-1 and Nrf-2	Wang et al. (2017a)
						(−) RAGE, MMP9; inflammatory factors expression	
Atrial interstitial fibrosis and atrial fibrillation	*in vivo*	SLI	Rats underwent center anterior descending coronary artery ligation	10, 20 and 40 mg/kg	5 weeks	(+) cardiac function	Qiu et al. (2018)
						(−) center atrial enlargement and P-wave duration; atrial hypertrophy; TXNIP/NLRP3 inflammasome/IL-1β; IL-18 signal pathway; BNP, IL-6, CRP, and TGFβ1	
Cerebral vascular diseases	*in vivo*	SLI	I/R rat Model	21 mg/kg	24, 48, 72 h	(+) ZO-1 expression; BBB function	Zhao et al. (2019a)
						(−) brain leakage of Evans blue; phosphorylation of ERK1/2 and Akt	
Diabetes and hyperglycemia	*in vivo*	DSI	Fed a high sugar and fat diet mice	6 g/kg	24 weeks	(+) HO-1	Zhou et al. (2020)
						(−) KLF10 upregulation; ROS generation	
Spinal cord ischemia	*in vivo*	PL	Acute spinal I/R injury was conducted by aortic occlusion	50 mg/kg	2 days	(+) motor function	Tian et al. (2015)
						(−) spinal infarction volume; Cdk5 and p25 activities	
Ischemic heart disease	*In vivo*	PL	Isoproterenol-induced myocardial infarction mice	40 mg/kg	5 days	(+) ventricular wall infarction	Li et al. (2018b)
						(−) typical ST segment depression; incidence of mortality; levels of myocardial injury markers; inflammatory milieu; TNF-α; IL-1βandIL-6	
Ischemic heart disease	*In vivo*	HJTI	Myocardial ischemia model	2, 4 mL/kg	7 days	(+) ATP content; LC3-II; beclin	Zhang et al. (2017)
						(−) Oxidative Stress; apoptosis rate; caspase 3 expression; Bcl-2/Bax ratio; phos-ERK; phos-AKT	
Diabetic angiopathies	*In vitro*	HJTI	HG-stimulated A7r5 cells	10, 20, 40, 80, 160, or 320 mL/L	48 h	(+) p53; cleaved caspase-3; Bax/Bcl-2 ratio	Fan et al. (2019)
						(−) pAKT; MMP9; PCNA	
myocardial infarction	*In vivo*	SGMI	Myocardial ischemia-reperfusion (MIRI) injury	6, 12 mL/kg	4 days	(+) Bcl-2; VEGF	Liu et al. (2018b)
						(−) myocardial apoptosis; Bax; caspase 3	
Extremity ischemia-reperfusion injury	*in vivo*	MLN	Posterior limb I/R injury rabbits	1.5 mL/kg	24 h	(+) SOD activity	Wang and Tian (2014)
						(−) levels of 8-iso-PGF2a	
Ischemic heart disease	*in vivo*	SXNI	MIRI model	4.38, 8.75, 17.5 mg/kg	3 days	(+) the activity of antioxidant enzymes	Wang et al. (2019b)
						(−) infarct size of myocardial tissue; myocardial enzyme and TnI levels; myocardial damage; MDA level; GRP78, CRT, CHOP, and caspase-12 expression levels; inflammatory cytokines; procoagulant molecules; TLR4/NF-κB expression	
Ischemic myocardial infarction and ischemic stroke	*in vivo*	SXNI	MIRI model	2.5 mL/kg	24 h	(−) cerebral infarction area; cerebral edema; TWEAK; Fn14	Xiao et al. (2019)
Stroke	*in vivo*	SXNI	MCAO model	3 mL/kg	7 days	(+) survival rate	Li et al. (2020c)
						(−) cerebral infarction and edema volume; G-csf; MAC-1; E-selectin; MAC-1	
Acute myocardial infarction	*in vivo*	SXNI	MIRI model	12.5 mL/kg	24 h	(+) cardiac function; mitochondrial function	Li et al. (2019c)
						(−) infarct size	
Stroke	*In vitro*	SLI + XSTI	Oxygen-glucose deprivation/reperfusion (OGD/R) injury model	3.125; 6.25; 12.5; 25; 50 μg/mL	24 h	(+) TEER; expression of tight junctions (TJs) between cells; stabilize the basement membrane (BM) composition	Yuan et al. (2021)
						(−) permeability of Na-Flu; Ang-2; VEGF	
Stroke	*in vivo*	SLI + XSTI	MCAO/R	XST 100 mg/kg + SLI 21 mg/kg	3 days	(+) regional cerebral blood flow; SOD; CAT; GSH; Nrf-2, HO-1, NQO-1; the nuclear translocation of Nrf-2	Wang et al. (2018b)
						(−) neurological deficit scores; infarct volumes; the activation of both microglia and astrocytes in the hippocampus; MDA; ROS; Keap1	
Myocardial ischemia-reperfusion injury	*In vitro*	SMI	H_2_O_2_-induced oxidative stress model of cardiomyocytes	0.2, 1 and 5 μL/mL	12 h	(+) SOD; GSR; CAT; P-Akt	Zhu et al. (2019)
						(−) proliferation arrest and apoptosis; ROS; NADH; MDA; the overloads of cytoplasmic Ca^2+^ and mitochondrial Ca^2+^; P-ERK1/2	
Ischemic myocardial infarction and ischemic stroke	*In vivo*	SXNI	Cerebral and myocardial I/R	2.5, 12.5 mL/kg	24 h	(+) cardiac function and coronary blood flow; myocardial infarction area	Lyu et al. (2018)
						(−) LDH, AST, CK-MB, and CK	
Ischemic stroke	*In vivo*	SXNI	Cerebral I/R model	3 mL/kg	7 days	(−) hippocampal neuronal apoptosis; the activation of Caspase-3 protein; Cleaved-Caspase-3	Lyu et al. (2018)
Ischemic stroke	*In vitro*	SXNI	HT-22 apoptosis caused by OGD/R	200 μg/mL	36 h	(−) the apoptosis rate; Bax and Cleaved-Caspase-3	Lyu et al. (2018)
Stroke	*In vivo*	SXNI	MCAO model	3 mL/kg	28 days	(+) repaired brain injury; BDNF and TrkB	Li et al. (2021e)
						(−) reduced neuronal apoptosis; level of p-Erk and Creb; GFAP	
ischemic stroke	*In vivo*	SXNI	MCAO model	1.83 mL/kg	72 h	(+) NOS3	Cui et al. (2020)
						(−) cerebral infarct volume; PTGS2 and CASP3	
heart failure	*In vitro*	XMLI	H9C2 rat cardiomyocytes	0.75 mg/mL	30 min	(−) phosphorylation of ERK1/2, AKT, and GSK3β; GATA4 in the nucleus	Qi et al. (2017)
Epirubicin-induced cardiotoxicity	*In vivo*	XMLI	Rats were intraperitoneally injected with epirubicin	125, 250, 500 mg/kg	14 h	(+) cardiac function; PKB/Akt; PI3K; Bcl_2_	Li et al. (2016)
						(−) center ventricle dilatation; the accumulation of collagen; Mmp9; Tgfb1; cardiac-fibrotic remodeling; autophagy; accumulation of Beclin1 and autophagy-related 7; phosphorylated P38; Erk1/2	
Stroke	*In vivo*	XNJI	Cerebral I/R injury	5, 10, or 15 mL/kg	24 h	(+) Bcl2/Bax; p-PI3K/Akt; p-eNOS; NO	Zhang et al. (2018)
						(−) the scores of neurological deficits; cerebral infarct volume; attenuated neuronal impairments; leukoaraiosis; apoptosis	
Ischemic stroke	*In vivo*	XNJI	Cerebral I/R injury	10 and 15 mL/kg	24 h	(+) neurological scores and morphological changes; SIRT1	Qu et al. (2019)
						(−) cerebral infarct area; inflammatory mediator levels	
Ischemic stroke	*In vivo*	XNJI	MCAO	15 mL/kg	24 h	(+) survival percent; tight junction protein, occludin and ZO-1	Zhang et al. (2020b)
						(−) infarct area and ameliorate neurological deficits; leaking amount of Evans Blue; NLRP3; inflammatory response; BBB disruption and brain damage	
Stroke	*In vitro*	XSTI	H_2_O_2_-injured cardiac cells	80 mg/kg	7 days	(+) the activity of PDH; intracellular contents of acetyl-CoA and ATP	Zhao et al. (2017)
						(−) intracellular MDA release	
Persistent myocardial ischemia	*In vitro*	DHI	H9C2 cells treated with H/R	5, 10, 20, 40, 80, 100 μg/mL	24 h	(+) mitochondrial morphology with increased mitochondrial length; ATP levels and the oxygen-consumption rate (−) anti-apoptosis action; ROS generation; mitochondrial dysfunction with a decreased mitochondrial membrane potential	Zhang et al. (2020a)
Ischemic cerebrovascular disease	*In vivo*	DHI	MCAO	0.5, 1, and 2 mL/kg	24 h	(+) the brain function score; anti-apoptotic factor Bcl_2_; PI3K-Akt signaling pathway	Feng et al. (2020)
						(−) brain tissue cell apoptosis; Bax, and Bim; apoptotic gene p53	
Ischemic heart disease	*In vivo*	DHI	Myocardial infarction model	1.5 mL/kg	28 days	(+) MSC survival rate and cardiac function; CXCR4; SDF-1	Chen et al. (2018)
						(−) myocardial infarct size; VEGF	

## Anti-platelet aggregation

DHI can inhibit inflammation and platelet aggregation, reduce immune response and peroxidation, and protect vascular endothelium and organ function, thus preventing and treating cardiovascular diseases (Zou et al., 2018; Bi et al., 2019). DHI can inhibit blood lipid levels and platelet aggregation rate in rats with hyperlipidemia. Meanwhile, thrombin time (TT), activated partial thrombin time (APTT), prothrombin time (PT), 6-K-PGF1a, PGI2, and PGE2 mRNA expression were significantly increased after DHI treatment, while the expression of TXA2 was significantly decreased (Fan et al., 2018). Based on path analysis and CMAP query of microarray data, researchers have found that anti-inflammatory response and anti-platelet coagulation are the main mechanisms of XSTI against stroke (Wang et al., 2015).

## Lipid profile and atherosclerosis

SXNI can effectively protect the brain and heart from I/R injury through the TNFRSF12a-mediated common pathway of atherosclerotic signaling and inflammatory responses (Lyu et al., 2018). DHI attenuates high-fat diet-induced atherosclerosis and macrophage lipid accumulation by modulating the PI3K/AKT pathway (Zhou et al., 2019). The effect of DHI on DC maturation and immune function induced by oxidized low-density lipoprotein is mainly through the activation of peroxisome proliferator-activated receptor γ (PPARγ) agonist signaling pathways (Liu et al., 2012).

## Anti-inflammatory

SLI attenuates inflammatory responses in BMMs and HUVECs. GHI can significantly improve brain I/R injury in rats, which may be achieved by inhibiting inflammation. GHI significantly reduces serum nitric oxide (NO), inducible nitric oxide synthase (iNOS), myeloperoxidase (MPO), interleukin-1b (IL-1b), tumor necrosis factor-α (TNF-α), and C-reactive protein (CRP) levels (Ai et al., 2017). Salvianolate treats myocardial infarction by inhibiting the TGF-β1/Smad2/3 and TXNIP/NLRP3 inflammasome signaling pathways (Qiu et al., 2018). XSTI, when combined with aspirin and clopidogrel, can protect rats from focal cerebral I/R injury by inhibiting oxidative stress and inflammation and regulating the NOX2/IL-6/STAT3 pathway. SXNI can prevent myocardial I/R injury by reducing oxidative stress, inflammation, and thrombosis (Zhu et al., 2021). SXNI can reduce the levels of inflammatory cytokines in serum, the levels of procoagulant molecules in plasma, and the expression of TLR4/NF-κB in rats (Wang R. et al., 2019). SXNI effectively protects the brain and heart from I/R injury through a common tnfrsf12a-mediated pathway involved in atherosclerotic signaling and inflammatory responses (Lyu et al., 2018). XNJI ameliorates cerebral I/R injury by inhibiting SIRT1-mediated inflammatory response (Zhang YM. et al., 2020).

## Anti-fibrotic and anti-apoptotic activities

Salvianolate reduces atrial fibrillation by inhibiting the TGF-β1/Smad2/3 and TXNIP/NLRP3 inflammasome signaling pathways in rats after myocardial infarction, thereby inhibiting atrial fibrosis (Qiu et al., 2018). DHI protects the heart of rats with myocardial infarction by resisting cardiomyocyte apoptosis and angiogenesis, and reducing myocardial fibrosis (Chen JR. et al., 2016). Pretreatment with SFI enhanced the expression of adenosine receptor A in a dose-dependent manner compared to that in the MI/R-post group (Wang et al., 2021b).

## Myocardial ischemia and cardiac hypertrophy

SXNI preconditioning has a cardioprotective effect on myocardial I/R injury, manifested as a reduced infarct size, improved cardiac function, and improved mitochondrial function (Li T. et al., 2019). SMI reduces apoptosis and enhances angiogenesis after myocardial I/R injury in rats. SMI-driven reduction in apoptosis is associated with changes in the ratio of Bcl-2 to Bax expression, whereas treatment-induced angiogenesis is associated with enhanced vascular endothelial growth factor A (VEGFA) expression (Liu X. et al., 2018). XSTI attenuates myocardial I/R injury by enhancing pyruvate dehydrogenase-mediated aerobic metabolism (Zhao et al., 2017). DHI attenuates isoproterenol-induced cardiac hypertrophy by modulating p38 and NF-κB pathways (Zhou et al., 2019).

## Cerebral ischemia

XSTI, when combined with freeze-dried sulfate injection, protects rats from focal cerebral I/R injury by inhibiting oxidative stress and the Nrf-2/Keap1 pathway (Wang FJ. et al., 2018). XNJI protects rats from cerebral I/R injury and alleviates blood-brain barrier damage by inhibiting the underlying mechanism of the NLRP3 inflammasome (Qu et al., 2019). DHI may reduce inflammation by maintaining the integrity of the brain-blood barrier and regulating TLR4-related signaling pathways, thereby effectively improving the prognosis of cerebral I/R injury (Qian et al., 2018).

## Regulating blood pressure and circulation

DHI reduces vascular remodeling and upregulates the kallikrein-kinin system in spontaneously hypertensive rats (Yang XH. et al., 2017). DHI prevents hypertension-induced renal injury by downregulating the expression of renal injury molecules and myoglobin in spontaneously hypertensive rats (Orgah et al., 2018). HHI can affect connective tissue growth factor; transforming growth factor-β and integrin expression can regulate pulmonary artery remodeling, thus affecting the wall thickness of the pulmonary and myocardial arterioles, which is conducive to the control of pulmonary hypertension (Chen et al., 2021).

## Clinical application of Chinese herbal injection in the cardio-cerebrovascular diseases

**FIGURE 4:**
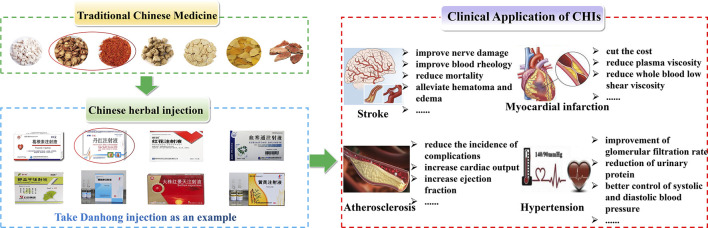
Clinical application of CHIs in cardio-cerebrovascular diseases, take Danhong injection as an example.

Most clinical trials of CHI for the treatment of cardio-cerebrovascular diseases have been conducted in China. In recent years, some scholars have collected relevant published randomized controlled trials for Bayesian network meta-analysis. We collected and summarized literature on the clinical application of CHIs to cardio-cerebrovascular diseases in recent years to provide a reference for the clinical use of CHIs in the treatment of cardio-cerebrovascular diseases, as shown in Figure 4.

## Stroke

The present study conducted a network meta-analysis of randomized controlled trials on the efficacy of CHI in the treatment of acute cerebral infarction, including 64 studies with 6,225 participants involving 15 TCM injections. In terms of apparent efficiency, DHI is most likely the best treatment option. In terms of improving nerve damage, SXNI has the highest probability of being the best treatment option (Huang et al., 2022).

In a randomized controlled trial of SYI in the treatment of acute cerebral infarction, the National Institute of Health Stroke Scale score in the SYI group decreased, and the hemorheological indices of red blood cell deformation and aggregation were significantly improved. The prothrombin time was increased, fibrinogen, TNF-α, and IL-1β levels were increased, and IL-6 levels were decreased (Li et al., 2015b). In a Bayesian network meta-analysis of randomized controlled trials of Danshen class injection in the treatment of acute cerebral infarction, including 157 randomized controlled trials with a total of 15,570 patients, the results showed that tanshinone IIA sodium sulfonate injection plus Western medicine is clinically effective; it is superior to other drugs in terms of neurological impairment and activities of daily living. DSI and SLI performed excellently in improving blood rheology (Liu S. et al., 2019).

A study conducted A meta-analysis of randomized controlled trials on the efficacy and safety of PI in the treatment of acute ischemic stroke. The meta-analysis identified 35 randomized controlled trials with a total of 3,224 participants. The results showed that PI was superior to the control drug in terms of clinical effectiveness, and the neurological deficit was significantly improved (Zheng et al., 2017).

A systematic review and meta-analysis of XNJI in the treatment of acute ischemic stroke showed that XNJ plus conventional treatment alleviated neurological deficits in acute ischemic stroke. Compared to DHI combined with conventional treatment, XNJ combined with conventional therapy reduces mortality (Tian et al., 2021; Wang L. et al., 2022). The researchers meta-analyzed 29 studies with a total of 2,638 patients. Compared with conventional treatment, XNJI is more effective, significantly reduces hs-CRP levels, enhances activities of daily living, and alleviates hematoma and edema (Ma et al., 2020). In a meta-analysis of the clinical efficacy of XNJI in the treatment of cerebral infarction, 53 randomized controlled trials involving a total of 4,915 participants, the results showed that compared to traditional treatment alone, XNJI can significantly improve the total effective rate, daily life enhanced ability, reduced infarct size, reduced neurological damage. XNJ can improve hemorheology and reduce whole-blood viscosity, plasma viscosity, and hematocrit. XNJ can also reduce cholesterol and triglyceride levels (Ma et al., 2017).

An efficacy and safety study of GDI in the treatment of ischemic stroke, with data from 39 trials including 3,182 ischemic stroke patients, showed that the general response of the neurological function in the conventional treatment and the GDI groups was significantly improved. The patients’ hemorheology and blood lipid indexes were also significantly improved after combined treatment (Wang YS. et al., 2017; Xue et al., 2019).

## Myocardial infarction and cardiomyopathy

Through a systematic review and meta-analysis of randomized controlled trials, some studies have compared the efficacy of DHI at different time points in the perioperative period of acute myocardial infarction. The analysis included 23 studies, all of which showed that the efficacy of the DHI was better (He et al., 2021). Some researchers have meta-analyzed GDI in the adjuvant treatment of angina pectoris, including 41 randomized controlled trials involving 4,462 patients. The combined application of GDI and Western medicine in the treatment of angina pectoris has a higher total effective rate and reduces plasma viscosity levels, fibrinogen, whole blood low shear viscosity, and whole blood high shear viscosity (Tan et al., 2018).

Some researchers have conducted a meta-analysis of 26 randomized controlled trials involving 3,447 participants to evaluate the therapeutic effect of XMLI on chronic heart failure. The results showed that XMLI plus conventional treatment improved the total efficacy rate. Compared with conventional treatment alone, XMLI combined with conventional treatment can increase left ventricular ejection fraction and 6-min walk distance, and reduce left ventricular end-diastolic diameter, serum brain natriuretic peptide, and N-terminal pro-brain natriuretic peptide (Lu et al., 2018). A randomized, double-blind, controlled study analyzed the effects of SMI on energy metabolism in patients with heart failure. The results showed that SMI improved patients’ energy metabolism compared to the trimetazidine and control groups, as evidenced by changes in serum-free fatty acid, lactic acid, pyruvate, and branched-chain amino acid levels (Wang SM. et al., 2020). This was a randomized, double-blind, multicenter, placebo-controlled clinical study on the efficacy and safety of SMI in the treatment of patients with chronic heart failure. The improvement in the form 36 hearth survey score and TCM syndrome score was better than that in the control group, the use of SMI treatment was well tolerated, and there were no obvious safety issues (Xian et al., 2016). In a cost-effectiveness analysis of SYI for the treatment of stable angina pectoris in China, SYI combined with conventional therapy was a cost-effective treatment option compared with conventional therapy for unstable angina pectoris (Xuan et al., 2018). A network meta-analysis of CHIs in the treatment of pulmonary heart disease, which compared the efficacy of seven CHIs with Western medicine in the treatment of pulmonary heart disease, included 118 randomized controlled trials with 10,085 patients. The results showed that Shenfu injection, SMI, and Shenqi Fuzheng injection combined with Western medicine may be the best treatment for pulmonary heart disease (Wang KH. et al., 2020).

## Atherosclerosis and coronary artery disease

A total of 53 qualified randomized controlled trials involving 6,401 patients were included in the treatment of acute coronary syndrome with DSI. The results showed that compared with Western medicine treatment alone, DSI combined with Western medicine treatment could significantly improve the curative effect (Guo SY. et al., 2020). A systematic review and meta-analysis of the efficacy of DHI combined with coronary revascularization in the treatment of acute coronary syndrome, included 14 studies involving 1,533 patients. DHI combined with surgical treatment of acute coronary syndrome can significantly improve acute coronary syndrome and reduce the incidence of complications after coronary intervention (Zou et al., 2018). SMI can effectively increase cardiac output, stroke volume, and ejection fraction in patients undergoing off-pump coronary artery bypass surgery, and improve the safety of anesthesia management (Liu QX. et al., 2018).

## Hypertension and hypertrophy

Some researchers have conducted a meta-analysis of tanshinone IIA sulfonate sodium injection in the treatment of hypertensive nephropathy, including 16 trials involving 1,696 patients. Tanshinone IIA sodium sulfonate injection combined with angiotensin receptor blocker (ARB) therapy is more effective than ARB monotherapy in regulating hypertensive nephropathy, manifested as improvement of glomerular filtration rate and reduction of urinary protein, cystatin, urinary immunoglobulin G, and urinary transferrin. In addition, combination therapy allows for better control of systolic and diastolic blood pressure (Xu JY. et al., 2019).

Some researchers have investigated the efficacy of DHI combined with antihypertensive drugs for the treatment of hypertensive nephropathy. The meta-analysis included 15 studies and the results showed that DHI combined with antihypertensive drugs was more effective in reducing microalbuminuria than antihypertensive drugs alone. The drug has the advantage of lowering systolic blood pressure, diastolic blood pressure, and serum creatinine (Li YZ. et al., 2020).

## Safety concerns, toxicity, and synergistic effects of CHIs

Although CHIs have been used clinically for many years and play an indispensable role in the treatment of cardio-cerebrovascular diseases, their adverse reactions have always been a focus of clinical attention. As the injection is a special dosage form, it is more likely to cause adverse reactions. CHIs are mostly complex systems containing many biologically active ingredients; therefore, the possibility of complex unforeseen effects will increase. Modern researchers have conducted extensive research on the safety of CHIs, including their quality control through chemical and biological methods. Through a pharmacokinetic study of CHIs, drug absorption, distribution, metabolism, and excretion *in vivo* were analyzed to evaluate their safety.

Adverse reactions to CHIs have always been the focus of attention. Scientific and reasonable quality control methods are crucial to ensure the stability and safety of their clinical application. Chemical and biological evaluation is the main of quality control methods for CHI. Because chemical components are critical to the efficacy of CHIs, component detection is the primary method for the quality control of CHI. For example, in the production process of SMI, its quality control index components were selected through the analysis of the component transfer process, and the quality control method of SMI injection was established (Zhao CX. et al., 2019). Quantitative analysis of SLI by 1H-qNMR and its quality control. qNMR can be used as a routine method for quality control of SLI and may be used for the quantification of diastereomers in other TCM preparations (Chen XL. et al., 2016). Rapid identification of the chemical constituents of DHI using liquid chromatography-mass spectrometry and precursor ion scanning enhanced liquid chromatography-tandem mass spectrometry (Li C. et al., 2019; Xu LL. et al., 2019). Rapid identification of chemical constituents in HJTI by liquid chromatography-quadrupole time-of-flight mass spectrometry (LC-Q-TOF-MS) can also be used to identify the chemical constituents of other *Rhodiola Rosea*-containing Chinese medicine formulations Element (Liu YN. et al., 2019). The phytochemical components of SXNI were identified using ultra-high-performance liquid chromatography-Q-precise mixed quadrupole orbital high-resolution mass spectrometry (UHPLC-Q-orbitrap HRMS) and nuclear magnetic resonance (NMR) techniques (Yu ST. et al., 2019). The detection of haptens in SXNI is based on human serum albumin fluorescence. A method for determining curcuminone, curcuminenol, curcuminenone, and gemmanone in XNJI was established based on a high-performance liquid chromatography-diode array (Pan et al., 2015).

Affected by the concept of “the higher the content of the index components, the better the quality,” there are still many drawbacks to a single chemical evaluation. It is easy to cause the post-marketing TCM products to be “qualified” according to the current quality standards, but cases of excessive biological activity affecting the clinical treatment effect occur occasionally. Therefore, biological evaluation is indispensable for the quality control of CHIs. For example, HHI detects mass fluctuations by chemical fingerprinting (ultra-performance liquid chromatography-tandem mass spectrometry) and bioassays (including cell-based bio-atlas assays and enzymatic assays) to screen out abnormal samples of HHI, and 33 compounds have been identified in HHI (Feng et al., 2018). In addition, *in vitro* anticoagulant activity evaluations of seven HHI samples from different companies through *in vitro* anticoagulant activity tests (Wang KH. et al., 2018). The efficacy and consistency of different batches of XSTI were evaluated based on bioactive chemical markers. First, the chemical structure of the XSTI was systematically characterized. Second, through *in vivo* validation based on the adjusted efficacy score, Panax notoginsenoside R1, ginsenoside Rg1, Re, Rb1, and Rd were identified as bioactive chemical markers for XSTI treatment of cardio-cerebrovascular diseases to assess the consistency between the batches (Yang ZZ. et al., 2017).

Owing to the particularity of the administration of CHIs, their safety has always been a concern for everyone. Therefore, pharmacokinetic research and analysis of the absorption, distribution, metabolism, and excretion of CHIs *in vivo* are important. Some researchers have developed the pharmacokinetics of ligustrazine after single and multiple intravenous injections of Shenxiong glucose in rats. After single and multiple intravenous injections of SXG, the pharmacokinetics of ligustrazine in rats showed a linear relationship with a half-life of approximately 35 min. Ligustrazine is easily distributed in organs with high perfusion and almost disappears from the organs 90 min after injection (Wang Q. et al., 2019). Pharmacokinetic study of salvianolic acid and ligustrazine in rat plasma after intravenous administration of DLI. The results showed that the elimination half-life (t_1/2_), AUC_0-t,_ and C_o_ in the tanshinol group were 30%–40% higher than those in the tanshinol ligustrazine injection group (Jiao et al., 2020). Some researchers have investigated the distribution kinetics of puerarin in the hippocampus of rats treated with puerarin injection after acute focal cerebral ischemia. The AUC of puerarin in the embolic hippocampus (AUC_0–120min_) was higher than that in the normal hippocampus. The average dwell time was higher than that of a normal hippocampus (Kong et al., 2019).

The content and pharmacokinetic analysis of borneol and muskone after the intravenous administration of XNJI in rats were determined by GC-MS/MS. At 8 and 1.5 h after intravenous injection of XNJ, the concentrations of borneol and muskone were 10 and 2.5 ng/mL (Song et al., 2017). Some researchers have also identified biologically active anti-angiogenic components targeting tumor endothelial cells in SMI through multi-dimensional pharmacokinetics, and protopanaxadiol (PPD) ginsenoside (Rb1, Rb2, Rb3, Rc, and Rd) concentrations were higher than those of protopanaxadiol (Rg1 and Re) and oleanane types (Rb1, Rb2, Rb3, Rc, and Rd). Among the PPD-type ginsenosides, Rd showed the highest concentration in tumors and TECs after repeated injections. *In vivo* bioactivity results showed that Rd inhibited neovascularization in tumors, normalized tumor vascular architecture, and enhanced the antitumor effect of 5-fluorouracil in xenografted mice. Furthermore, Rd inhibited endothelial cell migration and tube formation *in vitro*. In conclusion, Rd may be an important active form that exerts antiangiogenic effects on tumors after SMI treatment (Zhong et al., 2020).

Re-evaluation of the post-marketing safety of CHIs is also important to ensure the safety of clinical medication. Some researchers have evaluated the factors influencing suspected allergic reactions and systemic adverse reactions after SXNI. A randomized controlled study and cohort study were conducted on adverse drug reactions to SXNI using a computer database. When SXNI was used in combination with the chemical drugs, the adverse reaction rate was 4.36%. The incidence of allergic reactions to SXNI is also affected by the drug, treatment time, single dose, indications, and off-label use (Wang C. et al., 2018).

## Perspectives and challenges

The theory of TCM is summed up with long-term human experience, and its basis is oral or external administration. CHI has changed the traditional administration of TCM, which may produce new pharmacological effects or adverse reactions. In most cases, traditional medicine has limited guidance on the compatibility and proportion of CHIs. In addition, CHIs have the characteristics of a single component and multiple components, and exist as a single herb or prescription. Most CHI herbal studies are unclear, and pharmacology, pharmacokinetics, adverse reactions, and mechanisms of action research are not sufficiently thorough. In recent years, based on the multi-component and multi-target characteristics of CHIS, researchers have revealed the material basis of the efficacy of CHIS and its mechanism of action in treating diseases through network pharmacology, metabolomics, transcriptomics, and other technologies (Wang Z. et al., 2021). In addition, a large sample multicenter clinical trial of CHIS is conducive to ensuring the safety and effectiveness of its clinical application (Jiang et al., 2019; Cao et al., 2022; Yan et al., 2022).

Research on pharmaceutical preparations for CHIs is scarce. The technical requirements set standardized requirements for the pharmaceutical research content of CHIs, such as raw materials, excipients, preparation technology, and quality standards. Compared with oral TCM preparations, the quality of raw materials for CHIs should be higher, and the medicinal parts, origin (including origin processing), harvest season, storage conditions, and production of raw materials should be fixed. In terms of the preparation process, developers should fully explain the rationality of the process and comprehensively consider the impact of the process on the safety, effectiveness, and quality controllability of CHIs.

CHIs should have higher quality standards to ensure the safety and effectiveness of clinical use; therefore, quality research is very important in the research and development process of CHIs. Quality research includes literature research, chemical composition research, qualitative and quantitative analysis methods, and biological quality control methods. The quality control items of CHIs should consider the injection characteristics and sensitively reflect changes in drug quality (Wang N. et al., 2018; Tu et al., 2021). It is important to establish the pharmacodynamic material basis of CHIs and to rapidly detect harmful components to ensure safety and effectiveness (Bu et al., 2018; Zang et al., 2018).

## Conclusion

Compared with TCM preparations, CHIs avoid degradation of the gastrointestinal tract and the first-pass effect of the liver during traditional administration. The clinical application of CHI is more convenient, and its onset is faster. CHIs have been used clinically for many years and have played an important role in the treatment of acute and severe cardio-cerebrovascular diseases. However, some adverse reactions occur as a result of its complexity and deficiencies in development and production. These issues can be addressed by expanding the scope of our research. Through research, the main components of the injection can be determined and quality control oversight can be carried out so that the quality standard can be controlled. At the same time, further research on the mechanism of action and pharmacokinetics can reveal the scientific connotation of CHIs. In addition to standardizing clinical use and strengthening supervision, CHIs have broad application prospects.
